# APL@voro—interactive visualization and analysis of cell membrane simulations

**DOI:** 10.1093/bioinformatics/btad083

**Published:** 2023-02-08

**Authors:** Martin Kern, Sabrina Jaeger-Honz, Falk Schreiber, Bjorn Sommer

**Affiliations:** Department of Computer and Information Science, University of Konstanz, Konstanz 76484, Germany; Department of Computer and Information Science, University of Konstanz, Konstanz 76484, Germany; Department of Computer and Information Science, University of Konstanz, Konstanz 76484, Germany; Faculty of Information Technology, Monash University, Clayton, VIC 3800, Australia; Royal College of Art, School of Design, London SW7 2EU, UK

## Abstract

**Summary:**

Molecular dynamics (MD) simulations of cell membranes allow for a better understanding of complex processes such as changing membrane dynamics, lipid rafts and the incorporation/passing of macromolecules into/through membranes. To explore and understand cell membrane compositions, dynamics and processes, visual analytics can help to interpret MD simulation data. APL@Voro is a software for the interactive visualization and analysis of cell membrane simulations. Here, we present the new APL@Voro, which has been continuously developed since its initial release in 2013. We discuss newly implemented algorithms, methodologies and features, such as the interactive comparison of related simulations and methods to assign lipids to either the upper or lower leaflet.

**Availability and implementation:**

The current open-source version of APL@Voro can be downloaded from http://aplvoro.com.

## 1 Introduction

Cell membranes are central biological structures that separate the interior of a cell from the outside, thereby protecting the cell from its environment (Campbell, 2002). They consist of a lipid bilayer and contain various membrane proteins and small molecules. The properties of a cell membrane can change over time, for example, during different stages of the development of the cell or due to changes in its environment. Molecular dynamics (MD) simulations of cell membranes are a common approach to understand membranes and related processes. This includes using MD simulations to understand and prioritize cell-penetrating peptides (Tran *et al.*, 2021), cell membrane pore sealing (Zhang *et al.*, 2019), membrane permeability (Venable *et al.*, 2019) and many more processes.

Software tools for the analysis of cell membrane simulations play an important role when trying to understand the simulations. Examples are GridMAT-MD (Allen *et al.*, 2009), FATSLiM (Buchoux, 2017), MEMBPlugin (Guixà-González *et al.*, 2014), MemSurfer (Bhatia *et al.*, 2019) and APL@Voro (Lukat *et al.*, 2013). GridMAT-MD, like most tools, is a command-line tool that can calculate *membrane thickness* (*MT*) and *area per lipid* (*APL*) using algorithms similar to those used in APL@Voro. FATSLiM, another command-line tool, offers advanced algorithms to handle strongly curved membranes and vesicles. The output of both tools can then be visualized as 2D graphs with external packages like *Xmgrace* or Matplotlib (Hunter, 2007), but they do not provide direct internal visualization of membrane structures. To visualize the membrane itself additional software is required, such as VMD (Humphrey *et al.*, 1996) or UCSF Chimera (Pettersen *et al.*, 2004). MEMBPlugin is a plugin for VMD that can calculate APL, MT, order parameter, APL distribution and cholesterol tilt angle distribution. MEMBPlugin offers a user interface and includes the option to plot generated data. MemSurfer is a Python API that uses 3D point coordinates for Delaunay triangulations and surface parameterization to represent membrane surfaces. This procedure gives the user direct access to the membrane surface to enable calculations, such as MT and APL. The visualizations are however non-interactive which limits the exploration of the results. APL@Voro is developed with the goal of offering a software that unifies the analysis, interactive visualization, exploration and comparison of multiple membrane simulations. Table 1 contains a comparison of the different tools and their features.

**Table 1.** A comparison of different tools and their features

Category	Feature	MDAnalysis	FATSLiM	GridMAT-MD	MEMB-Plugin	MEMSurfer	APL@Voro
Membrane types	Flat membranes	✓	✓	✓	✓	✓	✓
	Curved membranes	✓	✓	✗	✗	✓	✗
	Vesicles	✓	✓	✗	✗	✗	✗
Comparisons	Multiple membrane comp.	✗	✗	✗	✗	✓	✓
Access	GUI	✗	✗	✗	✓	✗	✓
	Command line	✓	✓	✓	✓	✗	✓
	API	✗	✗	✗	✗	✓	✗
Visualizations	Voronoi	✗	✓	✗	✓	✓	✓
	Internal plotting	✓	✗	✗	✓	✗	✓
Interactions	Mouse interaction	✗	✗	✗	✓	✗	✓
	Linked views	✗	✗	✗	✗	✗	✓
	Interactive molecule selection	✗	✗	✗	✓	✗	✓

tick: supported by the tool; cross: not supported by the tool.

## 2 Methods and implementation

APL@Voro uses data obtained from GROMACS simulations (Abraham *et al.*, 2015) which are processed and then visualized for the analysis. It supports .PDB, .NDX, .XTC and .TRR formats. Other formats can be converted by using external tools such as MDAnalysis (Gowers *et al.*, 2016; Michaud-Agrawal *et al.*, 2011).

The data processing can be separated into the following steps:

1. leaflet detection,
2. Delaunay triangulation for each leaflet,
3. use of Delaunay triangulation to insert non-membrane atoms (e.g. protein) and calculate membrane thickness,
4. Voronoi diagram construction from Delaunay triangulation, and
5. use of Voronoi diagram to calculate APL.

**Fig. 1.**
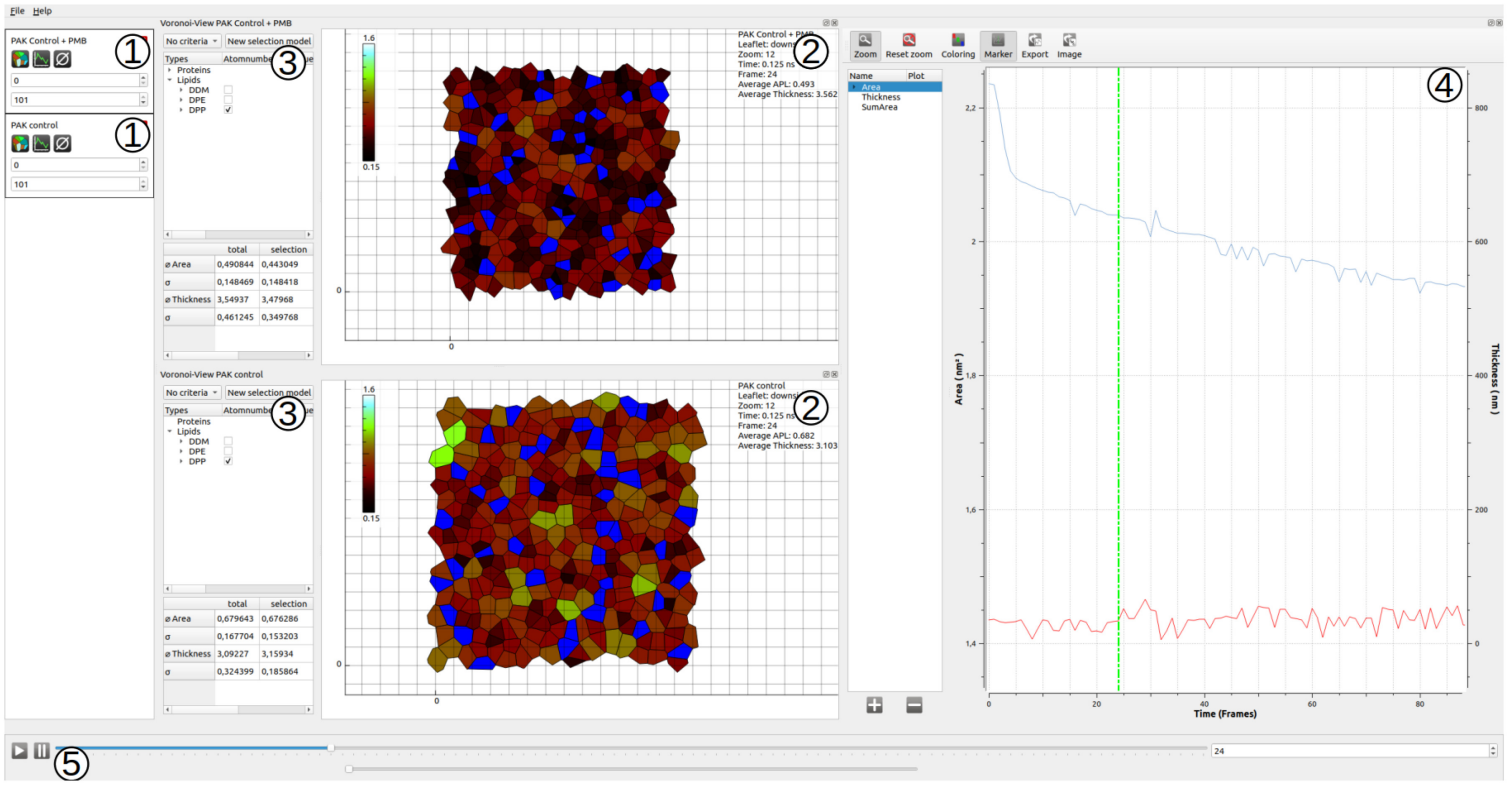
APL@Voro with two different membrane simulations. (1) Local simulation control panels which are used to open new views and synchronize simulations, (2) Voronoi views visualizing the APL using the linear optimized color scale (LOCS) (Herman and Levkowitz, 1992) (maximum: White—1.6 nm^2^, minimum: Black—0.15 nm^2^) and the blue cells represent user-selected areas (selected in 3., DPP), (3) selection panel with detailed information on each lipid, including area, local thickness and the key atom position, (4) 2D plotting view showing the average APL of the outer leaflet over time for both simulations and (5) global simulation control

The new version of APL@Voro is now capable of loading multiple simulations to compare them side by side. Each imported simulation appears in a list from where views can be opened. New views appear in a docking area that can be arranged by the user. Figure 1 shows an example of two imported simulations. Each simulation has a Voronoi view showing the lower leaflet. The 2D view contains the average area per lipid of the outer leaflet over time for both simulations. APL@Voro can synchronize simulations along frames which can be used to line up certain events (e.g. a substance entering the membrane) or account for different simulation lengths or time steps.

APL@Voro originally detected leaflets only by estimating the orientation of lipids (Lukat *et al.*, 2013). This approach is imprecise in some cases, e.g. for complex lipids such as lipid A or around membrane proteins. The newly added position-based approach fits a surface to the membrane using polynomial regression and uses the position of key atoms relative to that surface to assign lipids to a leaflet. Its runtime complexity is $O(nd^{2})$, where *n* is the number of lipid atoms, and *d* is the degree of the polynomial. This approach is in practice a bit slower than the orientation-based method (around 4%), but it proved more reliable.

The trajectory needs to be loaded into computer memory to enable responsive interactions, requiring a decent amount of memory space. Therefore, the new version allows the user to exclusively load relevant parts of a trajectory, as well as skip frames in order to manage memory usage. Loading 300 frames of a membrane with 23 764 atoms takes an average of 3.5 s on a system running Ubuntu 20.04 with a AMD Ryzen 7 5800X CPU and 32 Gb of 3200 MHz RAM.

The Voronoi view has been overhauled. The legend has now a fixed size and will be displayed in the top left corner. The top right corner is used to display additional information on the visualized leaflet. Also the grid rendering has been improved, the view can be dragged around by holding the middle mouse button. Hovering over a Voronoi cell will display information on the associated lipid next to the pointer. The original color scale options (rainbow or none) have been extended by three other color scale options.

APL@Voro can also be used as a pure command-line tool. Due to a lack of interactivity, there is no need to load trajectories into memory. Using the command-line functions will now only load one frame for analysis at a time to save memory.

To summarize the previous paragraphs, APL@Voro 3.3.3 offers the following new features in comparison to the old APL@Voro version:

- load multiple simulations for side-by-side comparison,
- frame synchronization,
- position-based leaflet detection,
- overhauled Voronoi view: improved visualization, easier navigation, larger area,
- possibility to load only parts of the trajectory,
- additional color scales (rainbow, LOCS, heated object and linear grey),
- optimization of memory usage (i.e. code optimization and 3D view removed) and
- optimized memory usage (by limiting the amount of memory used during parsing) when using console.

## 3 Usage

APL@Voro calculates APL and MT and creates an interactive visualization of membrane simulations. The average APL is related to other membrane properties like acyl chain ordering, compressibility and molecular packaging (Patra *et al.*, 2003). Changes in the average APL and MT, which are strongly related (Moradi *et al.*, 2019), can be indicative of various processes taking place in the membrane, e.g. phase changes.

APL@Voro visualizes membrane leaflets as a Voronoi diagram in the so-called Voronoi views where APL, MT and lipid neighborhood can be mapped to one of several color scales. Colors can also be manually assigned to lipid types. The Voronoi view is complemented by a table that contains detailed information on each lipid as well as averages for the whole leaflet and the current selection (see Fig. 1(3)). The Voronoi view can reveal lipid rafts, protein aggregation, local anomalies, etc. The user can define a selection based on a combination of conditions such as lipid type, MT, APL and neighbors. This can be used to track lipids with certain properties, e.g. lipids that are in the gel phase. These selections are also used for 2D views where the user can plot the average membrane thickness and area per lipid for all open simulations over time (Fig. 1(4)).

The results can be exported in various ways for further analysis: Voronoi views and 2D views as image file, simulation frames as either .TXT or .XML file, and 2D plots as .XVG file.

APL@Voro is available as executable versions for Linux as well as Windows. For Mac OS X (including M1 chipset), Windows versions can be used via CrossOver (https://www.codeweavers.com/). In the future, we are planning to provide native Mac OS X versions as well.

## 4 Discussion and future development

APL@Voro provides analysis and interactive visualization of cell membrane simulations. The new developments enable direct comparison of multiple membrane simulations within one session, better algorithms (e.g. for leaflet detection) and an updated intuitive graphical user interface. APL@Voro therefore allows for easy analysis of MD simulations and helps in exploring changing membrane dynamics, lipid rafts and membrane interactions of macromolecules. It is a good basis for MD simulation analysis but also allows for future developments such as exploring density profiles, diffusion coefficients, membrane curvature and deuterium order parameters.
